# The effect of virtual cognitive-behavioral sexual counseling on sexual function and sexual intimacy in pregnant women: a randomized controlled clinical trial

**DOI:** 10.1186/s12884-022-04932-4

**Published:** 2022-08-04

**Authors:** Manizheh Fathalian, Razieh Lotfi, Mahbobeh Faramarzi, Mostafa Qorbani

**Affiliations:** 1 Student Research Committee, Alborz University of Medical Sciences, Karaj, Iran; 2 Social Determinants of Health Research Center, Alborz University of Medical Sciences, Karaj, Iran; 3 School of Nursing and Midwifery, Alborz University of Medical Sciences, 1st Golestan- Eshteraki Boulevard- Baghestan, Karaj, Iran; 4 Infertility and Reproductive Health Research Center, Health Research Institute, Babol University of Medical Sciences, Babol, Iran; 5 Non-communicable Diseases Research Center, Alborz University of Medical Sciences, Karaj, Iran

**Keywords:** Pregnancy, Cognitive behavioral therapy, Sexual counseling, Sexual intimacy, Virtual counseling

## Abstract

**Background & aim:**

Pregnancy is associated with numerous physical and psychological changes and affects the sexual function of couples. Since the root of many marital problems lies in the quality of the relationship and sexual function, the present study investigates the effect of virtual cognitive-behavioral sexual counseling on pregnant women’s sexual function and intimacy.

**Methods & Materials:**

This study is a randomized clinical trial, and 80 pregnant women aged 18 to 35 years and in 16–24 weeks of pregnancy were assigned to two intervention and control groups based on randomized blocks from June 2021 to July 2021. The control group received routine prenatal care, but the intervention group, in addition to routine prenatal care, underwent virtual counseling with a cognitive-behavioral approach during eight sessions of 90 minutes. Data were collected using the Female Sexual Function Index (FSFI) and sexual intimacy questionnaire. SPSS software was used for statistical analysis.

**Results:**

The comparison of mean scores of sexual function and intimacy in the two groups before the intervention was not statistically significant with each other. However, after the intervention, the mean scores of sexual function and intimacy in the intervention group were significantly increased compared to the control group (*p* < 0.001). The effect size of the intervention was 0.52 for sexual function and 0.272 for sexual intimacy.

**Conclusion:**

Virtual cognitive-behavioral counseling can be used as an effective treatment to promote sexual function and intimacy of women during pregnancy.

**Trial registration:**

IRCT20161230031662N10. Registry date: 21/06/2021.

## Introduction

Pregnancy is one of the most sensitive and important periods in a woman’s life [1]. The biological, psychological, and social changes created during this period can lead to changes in sexual function and emotional relationships [2]. As a result, sexual dysfunction is common in pregnant women [3, 4], and studies have reported the prevalence of these disorders to be about 57 to 93% [1, 4]. In one study, more than 60% of women and more than 40% of their husbands reported decreased desire and sexual function during pregnancy [5].

Factors affecting women’s sexual function in pregnancy can be divided into biological, psychological, and interpersonal factors [3, 6]. During pregnancy, hormonal and physical changes, abdominal enlargement and breast tenderness, fatigue, and back pain can affect women’s sexual desire [7]. Psychological factors such as women’s self-concept of losing attractiveness to their partners, feelings of shame and guilt about sex and negative attitudes about sex during this period, fear of miscarriage, fetal harm, and preterm delivery are all among the factors that reduce sexual desire and reduce emotional and loving relationship from the spouse and cause anxiety and lack of self-confidence in the mother [8].

Sexual behaviors and attitudes of individuals during pregnancy are influenced by tradition, cultural values, society, wanted or unwanted pregnancy, the type of relationship with the spouse, religious beliefs, and medical conditions [1, 7]. Studies show that men experience their first extramarital sex during their wives’ pregnancy, which may be due to the fact that their sexual and emotional needs are not met [7, 9]. This causes conflict in couples’ relationships, and just when they need more than ever to be close to each other, they become cold and quarrelsome [10].

Sexual intimacy as an important factor in marital relationships is the need to share, contribute and express sexual thoughts, feelings, and fantasies with the spouse [11, 12]. Intimacy in sexual relationships is directly related to satisfaction and quality of married life [13, 14], leading to more physical and mental health among couples. Sexual intimacy is associated with longer telomere length [15]. Studies suggest that Women’s sexual intimacy in the couple relationship may promote higher sexual satisfaction and sexual function [16]. There is a correlation between sexual intimacy and sexual dysfunction. Indeed, the lack of intimacy may be associated with sexual dysfunction and emotional problems. Moreover, the most important cause of extramarital sexual relationships is seeking to experience personal and sexual intimacy [17].

Sexual dysfunction is successfully treated with various methods such as sex therapy, psychological education, couple therapy, and Cognitive-behavioral Therapy (CBT) [3, 18]. Today, one of the current methods used to treat sexual dysfunction is CBT [19]. CBT includes identifying and correcting thought patterns, teaching communication skills, teaching distorted thoughts, dealing with sexual problems, as well as conflict resolution methods that lead to intimacy between couples [20, 21]. Cognitive-behavioral techniques for the treatment of female sexual dysfunction include increasing sexual awareness, training sexual fantasies and helping individual concentration and attention to pleasurable sexual stimuli, reducing anxiety about sexual activity through a combination of methods of sensate focus exercises, attention concentration skills, exposure, and cognitive reconstruction and problem-solving ability [21–23].

Cognitive-behavioral counseling is an effective treatment for managing dyspareunia, improved sexual function, and marital and psychosocial adjustment [24]. The effectiveness of CBT was demonstrated in various studies [25, 26]. However, in a study in Iran, sexual counseling with a cognitive-behavioral approach could not improve the sexual function of pregnant women [27]. In another study, it was reported that the sexual function of pregnant women in the group which received education did not show a significant difference compared to the control group [28]. Although the efficacy of CBT in treating the sexual problems of pregnant women was studied in many studies, many women do not seek treatment for their sexual problems yet because of social stigma [29]. Many barriers will be removed to seeking help for sexual problems if this therapy is available virtual [30]. Findings support increased access and a cost-effective alternative to existing face-to-face CBT for sexual problems [31].

The Internet plays a significant role in exchanging information in our daily lives. Electronic information is shared in various ways, including audio and video activities, the Internet, multimedia, virtual meetings, and video conferencing [32]. Virtual counseling is a type of telemedicine that includes any treatment a person seeks through an electronic device [33]. The benefits of virtual counseling include: saving time and money, easy access, increased speed of learning, taking advantage of teamwork, independence of time and place, up-to-date information, elimination of unnecessary traffic, easier self-disclosure of patients, privacy, being more attractive to learners, the ability to browse content, videos, and images [34, 35]. Due to the high effectiveness and efficiency of Internet-based CBT in some mental disorders, this method is in the first place compared to face-to-face CBT [31]. Internet-based CBT can help people deal with their problems and relapses [36].

Besides, crises such as COVID-19, which may coexist with the world for years, can affect the sexual behavior of pregnant women [37]. Standard public health measures, including quarantine, and social distancing to control the disease, have changed the relationship between therapists and patients. COVID-19 has changed social lifestyles, and the growing need for virtual counseling using distance health education seems essential [38]. Therefore, this study aimed to assess the effects of virtual sexual counseling based on CBT approach on the sexual function and intimacy of pregnant women.

## Materials and methods

### Design

This study is a randomized controlled clinical trial performed with intervention and control groups. Samples were selected from health centers in Alborz province. Health centers in Alborz province include two health centers in the west and east district. Among them, ten centers (five centers in each district) were selected randomly after determining the list of the active centers with an adequate number of the pregnant coverage (existing at least ten records of pregnancy).

Convenience sampling was performed from June 2021 to July 2021 using patients’ electronic records in a way that patients with inclusion criteria were selected. Then, by calling the qualified pregnant women, they were asked to complete the informed consent form if they were willing to participate in the study, and thus 80 qualified mothers were selected. A four-block randomization method was used for randomization. Block randomization was performed using the website www.sealedenvelope.com. Also, using the same tool for random concealment, unique codes were assigned to each assignment to maintain the random assignment concealment. A colleague not involved in the study conducted randomization and concealment. Thus, 80 participants were randomly and equally divided into two groups of intervention (*n* = 40) and control (*n* = 40).

This study was approved by the ethics code IR.ABZUMS.REC.1400.023 in the ethics committee of Alborz University of Medical Sciences and was registered with the code IRCT20161230031662N10 in the Clinical Trial Registration Center of Iran. The registration date was 21/06/2021.

### Study participants

This study included 80 married pregnant women aged 18 to 35 years. Inclusion criteria were: Primipara, gestational age of 16 to 24 weeks, single pregnancy, healthy and wanted pregnancy, sexual function score less than 28 in FSFI, Iranian nationality, having a personal cellphone, having the literacy of using the Internet to communicate, not using drugs that affect sexual function (such as SSRIs, antihistamines …), not having sexual dysfunction in a spouse (self-declaration), having a monogamous cohabitation, not having a known severe mental illness and chronic physical illness, not having grief during 3 months before the study, not attending similar treatment sessions for the past 6 months. Exclusion criteria include intrauterine fetal death, a history of previous miscarriage, high-risk pregnancy such as (placenta previa, threatened abortion, the possibility of preterm delivery, vaginal bleeding, incomplete cervix, cerclage, pregnancy with the help of Assisted Reproductive Technology (ART), premature rupture of membranes, unwillingness to continue participating in the study or being on other treatment, the occurrence of any problems in pregnancy during the study that required medical intervention and the absence of more than two sessions in the consultation session.

**Fig. 1:**
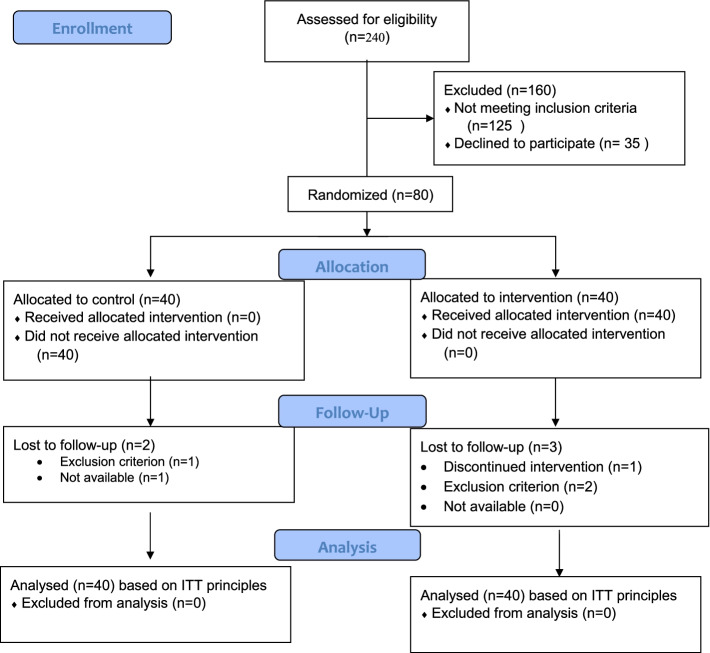
CONSORT 2010 Flow Diagram. ^×^ ITT (Intention to treat)

Measurement of the outcomes was performed by completing questionnaires online. These questionnaires were completed by both intervention and control groups at the beginning of the study and at the end of 4 weeks after the intervention. Two people from the control group and three people from the intervention group were excluded from the study during the follow-up. In the intervention group, three individuals were excluded from the study; one due to COVID-19 and hospitalization, and others due to abortion or non-participation in counseling sessions. Moreover, one person was excluded from the study in the control group due to abortion, and one person was excluded due to not completing the second phase questionnaire. The flowcharts of the study participants are shown in Fig. 1.

### Sample size

In this study, the number of the participants based on the study of Nezamnia et al. [26] considering α = 0.05 and β = 0.2 and the difference in sexual function score before and after in the two groups equal to 3.6 points and the standard deviation of the intervention group was 4.64. For the control group, it was 6.78, so the sample size in each group was determined to be 40 people, based on the following formula:

$$\mathbf{n}=\frac{{\left({\mathbf{z}}_{\mathbf{1}-\frac{\boldsymbol{\upalpha}}{\mathbf{2}}}+{\mathbf{z}}_{\mathbf{1}-\boldsymbol{\upbeta}}\right)}^{\mathbf{2}}\left({{\boldsymbol{\upsigma}}_{\mathbf{1}}}^{\mathbf{2}}+{{\boldsymbol{\upsigma}}_{\mathbf{2}}}^{\mathbf{2}}\right)}{{\left({\mathbf{Mean}}_{\mathbf{2}}-{\mathbf{Mean}}_{\mathbf{1}}\right)}^{\mathbf{2}}}$$

### Measurements

Data was gathered based on a three-part questionnaire including; demographic and individual characteristics, female sexual function index, and sexual intimacy questionnaire. Personal characteristics included; age, duration of the marriage, gestational age, job, education, and economic status.

### Female’s sexual function index (FSFI)

The sexual function index of Rosen et al. was used to assess women’s sexual function [39]. FSFI includes 19 questions that measure female sexual function in six independent areas of sexual desire, arousal, lubrication, orgasm, sexual satisfaction, and pain in the last 4 weeks. FSFI questions are scored from zero to five based on the Likert scoring system. Zero indicates a person who has not had any sexual activity in the last 4 weeks. A higher score indicates better sexual function. A score below 28 was considered a cut-off for sexual dysfunction [40]. The validity and reliability of this instrument were confirmed in Iran, and Cronbach’s alpha was determined to be 0.72 [41]. In the present study, Cronbach’s alpha was calculated to be 0.81.

### Sexual intimacy questionnaire

In this study, the sexual intimacy questionnaire was a modified form of Bagarozi Marital Intimacy Questionnaire [11], which its psychometric properties were examined in Iran [42]. This questionnaire contains 30 four-point Likert scale questions (always, sometimes, rarely, and never). According to this questionnaire, the lowest score for each person is 30, and the highest score is 120. A higher score indicates more sexual intimacy, and people who score less than the mean score are considered low sexual intimacy. The validity and reliability of this questionnaire have been investigated in Iran. Cronbach’s alpha coefficient was 0.81 [43]. In the present study, Cronbach’s alpha was calculated to be 0.84.

### Intervention

The intervention group participated in eight 90-minute group sexual counseling sessions held weekly. The content of sessions was designed based on virtual CBT and performed under the supervision of a clinical psychologist. The main researcher completed the CBT course. Virtual counseling content was implemented in the form of educational slides, text files, audio, and educational videos through Skyroom and WhatsApp. All the sessions were performed online on a virtual educational platform named Skyroom (an Iranian platform). On this site, effective interaction may be done, and participants can ask or answer the questions. Real-time audio and video connection were available. The contents were not just in self-led format. The first author presented all the materials in the class in the presence of two supervisors. After that, the recorded file of the course was uploaded on WhatsApp. Moreover, some written educational files were uploaded as reminders. About half of the time in the class was devoted to the participants’ discussion about their problems or concerns.

To assess the participants’ thoughts and beliefs, they were asked to say what they thought and felt about having sex during pregnancy. This way, most of the intervention time was related to the discussion. The content of the intervention is presented in Table 1 [8, 12, 13, 26, 44–51].

**Table 1:** Content of counseling sessions

Session	Training goal	Program content
**1**	Familiarity with the group and method of treatment, anatomy, and physiology, the sexual cycle of men and women	Establishment of professional communication, attracting cooperation, statement of meeting rules, the introduction of research goals and the treatment method, expression of treatment importance, familiarity with male and female genitals and sexual cycle
**2**	Investigating and explaining the areas of sexual function and its changes during pregnancy, training and practicing emotions and behavior and relaxation training, cognitive errors	Training of identifying emotions, thoughts, and behaviors, paying attention to the positive and negative aspects of couples’ relationships, the need to process positive sexual thoughts along with muscle relaxation during sexual intercourse, correction of cognitive errors, assignment of identifying self-negative thoughts - positive thoughts, concentration and practice of deep breathing and muscle relaxation
**3**	Continuing exercises to change dysfunctional sexual thoughts, defining intimacy and its dimensions, teaching how to establish intimacy, more spouse participation	Training techniques and activities for sexual intimacy, identifying and respecting spouse’s disparate interests, the effect of sexual security on couples’ relationships, understanding spouse’s needs and trying to satisfy it to increase intimacy, changing negative sexual attitudes by cognitive distortion, sensate focus, breathing and relaxation
**4**	Study of sexual dysfunctional feelings, thoughts, and behavior during pregnancy, the practice of sexual fantasies, study of conflict resolution methods	The impact of automatic negative thoughts on feelings and behavior, the study of conflict resolution methods, diagnosis and treatment of misconceptions, an increase of sexual information, self-reliance in identifying cognitive errors, teaching sexual fantasy
**5**	Practice recognizing cognitive errors, describing the pattern of sexual changes during pregnancy, practicing sexual fantasies	Expressing the importance of intercourse, factors that prevent proper sexual intercourse, focusing on sexual feelings and massaging the sexual areas, explaining the steps of problem-solving, at this stage the goal was a pleasure and sexual arousal without vaginal penetration, examining the effect of thoughts on sexual behavior
**6**	Logical alternatives, explaining the different positions of intercourse during pregnancy, relaxation exercises, and Kegel exercise	Logical thinking and alternatives to logical thoughts. Task: Logical alternatives in treatment model – evidence review, training how to have sex during pregnancy, teaching appropriate positions during pregnancy, relaxation and Kegel exercises during pregnancy, practice relaxation and Kegel
**7**	Assessing progress in pregnant mothers and assessing their beliefs, factors affecting sexual focus	Review of changes of sexual behaviors, discovering negative thoughts about sex and reviewing negative thoughts through cognitive distortion, foreplay, and romantic relationships, identifying irritable points in the body
**8**	Summarizing and reviewing training of sexual relations techniques and sexual concentration, prevention of recurrence	Summary and general review of trained materials and skills and answering questions, expressing participants’ experiences, determining follow-up time, Assignment: Cards of coping with prevention from recurrence

The primary outcome was sexual function, and the secondary outcome was sexual intimacy.

Exercises and assignments were given at the end of the sessions, including paying attention to the positive and negative aspects of couples’ relationships, identifying self-negative thoughts - positive thoughts, concentrating and practice of deep breathing and progressive muscle relaxation (PMR), Sensate Focus, focusing on sexual feelings and massaging the sexual areas, Logical thinking, discovering negative thoughts about sex, cards of coping with prevention of recurrence. And in the following sessions, assignments and the extent of people’s improvements were reviewed by looking at the assignments and reading them to ensure they were successfully done. The ambiguities and questions of the samples were answered.

#### Statistical analysis

Data were analyzed using SPSS software and described in the range of 95% with mean and standard deviation. The Chi-square test was used to analyze qualitative data and compare the percentage of participants in each group who scored more than 28 from FSFI. To evaluate the normality of the data, Shapiro-Wilk test was used, which had a normal distribution. Data were analyzed using paired t-test, independent t-test, and two-way mixed ANOVA. The significance level was less than 0.05. Using multiple imputation methods, the missing data were imputed in both groups, and the data were analyzed with the intention to treat (ITT) analysis approach.

## Results

The participants in the two groups of intervention and control had no statistically significant differences in demographic and obstetric characteristics in terms of the age of pregnant women, the duration of marriage, gestational age, level of education, occupation, and economic status. The results are shown in Table 2.

**Table 2:** Basic characteristics of the pregnant women in control and intervention groups

Variables	Intervention (***n*** = 40)	Control (***n*** = 40)	***p***-value^*^
Age (year)^a^	26.05 ± 4.40	26.53 ± 5.42	0.668
Duration of marriage (year)^a^	3.83 ± 2.17	3.93 ± 2.44	0.847
Gestational age (week)^a^	20.30 ± 3.16	20.18 ± 3.50	0.867
Job^b^			
Housewife	34 (85%)	33 (82.5%)	0.765
Employed	6 (15%)	7 (17.5%)	
Education^b^			
Up to Diploma	21 (52.5%)	23 (57.5%)	0.653
College/University	19 (47.5%)	17 (42.5%))	
Economic Status^b^			
Weak	15 (37.5%)	12 (30%)	0.156
Average	9 (22.5%)	17 (42.5%)	
Good	16 (40%)	11 (27.5%)	

^a^Data presented as mean ± SD

^b^Data presented as frequency (%)

^*^According to t-test (for continuous variables) and Chi-square test

At the beginning of the study, the mean score of sexual function in the two groups was almost equal, and no statistically significant difference was observed (*P* = 0.524). The results showed that in the intervention group, the mean and standard deviation of sexual function score before the intervention was 22.93 ± 3.80, and it reached 28.39 ± 2.65 after the intervention, which was a statistically significant change compared to the pre-test (*p* < 0.001). Also, after the intervention, a significant difference was observed in the two groups in the areas of sexual function (desire, arousal, vaginal lubrication, orgasm, satisfaction, and pain). The results are shown in Table 3.

**Table 3:** Comparison of mean scores of sexual function domains before and four weeks after the intervention in pregnant women in study groups

FSFI and domains	Group (***n*** = 40)	Before intervention	Four weeks after intervention	***p***-value		F	η^2^***
				Within-group^**^	Between-group^*^		
**Desire**	Intervention Control	3.43 ± 0.99 3.55 ± 0.6	4.1 ± 0.66 3.62 ± 0.58	*P* < 0.001 *P* = 0.544	*P* < 0.001	15.09	0.162
**Arousal**	Intervention Control	3.61 ± 1 3.43 ± 0.81	4.57 ± 0.64 3.50 ± 0.78	*P* < 0.001 *P* = 0.311	*P* < 0.001	30.20	0.279
**Lubrication**	Intervention Control	3.98 ± 1 4.16 ± 0.92	5.02 ± 0.60 4.23 ± 0.97	*P* < 0.001 *P* = 0.561	*P* < 0.001	24.55	0.239
**Orgasm**	Intervention Control	4.06 ± 1.19 4.11 ± 1.12	4.96 ± 0.80 4.17 ± 1.12	*P* < 0.001 *P* = 0.666	*P* < 0.001	11.98	0.133
**Satisfaction**	Intervention Control	4.27 ± 1.01 4.34 ± 0.90	5.13 ± 0.68 4.29 ± 1.02	*P* < 0.001 *P* = 0.621	*P* < 0.001	24.88	0.242
**Pain**	Intervention Control	3.57 ± 1.35 3.81 ± 1.04	4.57 ± 0.82 3.80 ± 0.95	*P* < 0.001 *P* = 0.886	*P* < 0.001	26.55	0.254
**FSFI**	Intervention Control	22.93 ± 3.80 23.40 ± 2.70	28.39 ± 2.65 23.62 ± 2.68	*P* < 0.001 *P* = 0.396	*P* < 0.001	83.96	0.520

Data presented as mean ± SD.

^*^According to two-way mixed ANOVA

^**^According to Paired t-Test

*** η^2^ **(**eta effect size)

Table 4 showed that sexual intimacy in the intervention group, the mean and standard deviation before the intervention were 93.80 ± 11.80. In the post-test, it reached 105.09 ± 9.43, which was a statistically significant change compared to the pre-test (*p* < 0.001).

**Table 4:** Comparison of mean scores of sexual intimacy before and four weeks after the intervention in pregnant women in study groups

Groups (***n*** = 40)	Before intervention	Four weeks after intervention	***p***-value		F statistic	ETA
			Within-group^**^	Between-group^*^		
**Intervention**	93.80 ± 11.80	105.09 ± 9.43	*P* < 0.001	*P* < 0.001	29.19	0.272
**Control**	94.43 ± 13.75	93.97 ± 13.66	*P* = 0.724			

Data presented as mean ± SD.

^*^According to two-way mixed ANOVA

^**^According to Paired t-Test

Before the intervention, FSFI scores of the participants were under 28. After the intervention, 57.5 participants in the intervention group scored higher than 28. However, only one participant in the control group scored more than 28, and 39 people remained lower than 28. (χ2 test; *p* = 0.000).

**Fig. 2:**
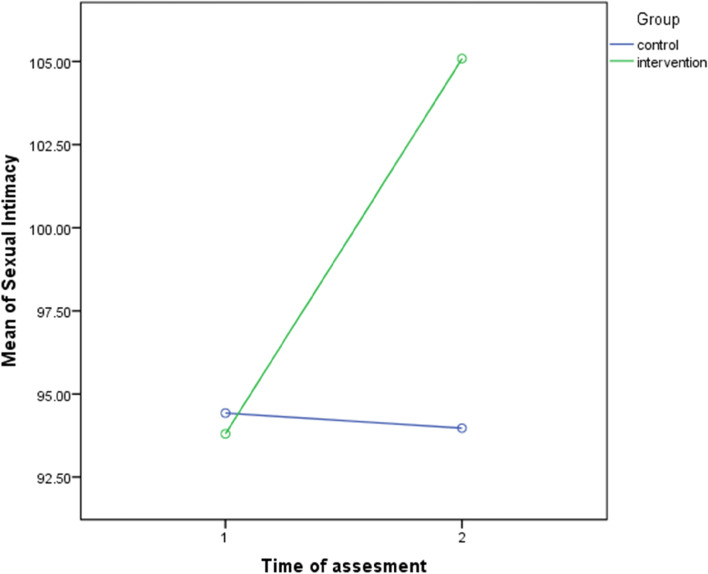
Sexual intimacy of the intervention and control groups before intervention and 4 weeks after intervention

Sexual intimacy of the intervention and control groups before the intervention and 4 weeks after the intervention are shown in Fig. 2.

## Discussion

This study showed that virtual cognitive-behavioral sexual counseling is effective in sexual function and intimacy of pregnant women. The results indicate that counseling improved sexual function in the intervention group by raising the mean score of sexual function above the cut-off point. Also, considering the size of the effect of the intervention on the sexual function of pregnant women (eta = 0.52), which is a strong effect, it means that virtual cognitive-behavioral sexual counseling is one of the most effective treatment methods to improve female sexual function.

According to the present study’s findings, a review study by Widman et al. (2018) aimed at investigating the effect of technology-based interventions on sexual knowledge and attitude showed that technology-based sex education increases sexual knowledge, strengthens positive sexual attitude improves healthy sexual behaviors [52]. Given that virtual interventions effectively change people’s attitudes, beliefs, and behaviors and improve their sexual function, it can be expected that barriers to face-to-face referral and access to the health care system [53, 54] should be eliminated to some extent.

One of the important aspects of the intervention is its durability. In the present study, the effect of the intervention was investigated up to 4 weeks after the intervention, while in another study by Myers et al. (2020), with similar findings and confirmation of the effect of Internet-based cognitive behavioral therapy on female low desire, the follow-up period was up to 12 months which indicates the long-term effectiveness of this intervention method [55]. Due to educational deprivation and misconceptions about sexual desires [56], it can be expected that many problems in marital relationships can be prevented by modifying and adjusting women’s beliefs about sexuality. This is because most women do not receive information about sexual issues during prenatal care [7]. In the study of Afshar et al., the importance of sex education was shown to positively affect the sexual life of pregnant women [57].

Negative attitudes and dysfunctional beliefs about sex in pregnancy and the frequency of intimacy or unreasonable interruption with a lack of understanding of the physical and emotional changes of this period and incorrect and inadequate information about couples about sex in pregnancy can weaken the emotional and loving relationship of couples, anxiety and lack of self-confidence and mental health disorders of couples [58]. The most effective and common treatment methods were the cognitive-behavioral approach through cognitive reconstruction techniques, reducing anxiety such as relaxation, providing sexual information, practicing sensate focus, and systematic desensitization [25]. Internet-based cognitive-behavioral therapy as a cost-effective treatment [31] has a high impact on sexual intimacy [47], sexual function and satisfaction [24, 59], body image of women with breast cancer, and sexual dysfunction [30]. They also reported that cognitive-behavioral therapy increases sexual desire, improves sexual arousal, reduces depression and discomfort during sex.

Exercises prescribed in CBT are not just physical acts; these sexual exercises can cause complex psychological processes in individuals. For example, pleasurable reactions are strengthened in sensate focus, reducing sexual tensions on both sides and increasing the couple’s emotional connection during these exercises. This treatment allows people to express their emotions easily and freely; it also reduces anxiety and facilitates communication. Eliminating guilt or fear of success and pleasure and replacing correct cognitions with dysfunctional cognitions can justify this therapeutic intervention.

On the other hand, in the study of Vakilian et al., who examined the effectiveness of the cognitive-behavioral sexual counseling approach on sexual function of pregnant women in Iran, counseling failed to cause psychological and biological changes. It did not improve the sexual function of participants [27]. One of the reasons for this difference is how the outcome variable is measured. Most studies in this field are based on comparing the mean score of sexual function. In contrast, in the study of Vakilian et al., the percentage of people who reached a mean score of the sexual function above 26 was set as the basis that although the mean score of sexual function in pregnant women in the intervention group increased compared to the control group. However, only 36.5% of participants reached a score above 26, which was in an inadequate range. However, increasing the mean sexual function of 36.5% of the participants is also a valuable finding. This finding seems satisfactory in pregnant women considering the biological and hormonal changes during pregnancy.

Table 4 indicates that counseling has improved sexual intimacy in the intervention group. The mean score of sexual intimacy has increased significantly. Also, considering the intervention’s effect size on the sexual intimacy of pregnant women (η^2^ = 0.272), which is a good effect, it can be said that virtual cognitive-behavioral sexual counseling is one of the effective treatment methods to improve female sexual intimacy.

Counseling and correcting insights about sex can improve couples’ relationships and increase intimacy, especially during pregnancy. One of the components of CBT is the presentation of sexual knowledge and sexual self-expression. When people trust each other and express their thoughts and feelings, they can establish an intimate relationship, which leads to positive feelings and increases couples’ sexual intimacy, quality of life, and sexual health [60–62]. A study that compared the effectiveness of group counseling sexual enrichment with a CBT approach in the presence of sexual intimacy in pregnant women stated that sexual cognitive-behavioral counseling had improved sexual intimacy in pregnant women in both online and face-to-face groups. However, the online counseling group reported more intimacy and sexual satisfaction, which can be beneficial in the COVID-19 pandemic [47]. In the present study, cognitive-behavioral counseling and the effectiveness of methods to increase sexual knowledge and self-expression have increased sexual intimacy in the intervention group. The mechanism of the cognitive-behavioral approach is strengthening positive exchanges, teaching communication skills, changing and modifying thinking patterns, changing distorted attitudes and beliefs, detecting annoying thoughts, overcoming sexual problems, and problem-solving methods that lead to increased satisfaction and intimacy [21, 23, 63].

One of the present study’s limitations was the impossibility of spouses’ active presence in all the sessions. Despite our interest in having spouses in all the sessions, we aimed their presence in one session due to their busy schedules. Moreover, this study did not assess some variables such as self-perception of sexual dysfunction, presence of sexual distress, and type of sexual activity. The researcher did her best to Mothers who did not have access to high-speed Internet could not attend the study. Also, due to the limited study time, it is suggested that a study be designed and conducted with a more extended follow-up period.

## Conclusion

As we could show that virtual cognitive-behavioral sexual counseling may improve sexual function and sexual intimacy of pregnant women, this approach can be implemented to promote the sexual health of pregnant women. However, more studies are needed to evaluate the feasibility of performing cognitive therapy intervention virtually to discover and eliminate its implementation problems in different groups of pregnant women from other socio-economic classes.

## Data Availability

The datasets used and analyzed during the current study are available from the corresponding author on reasonable request.
